# Emerging strategies to target RAS signaling in human cancer therapy

**DOI:** 10.1186/s13045-021-01127-w

**Published:** 2021-07-23

**Authors:** Kun Chen, Yalei Zhang, Ling Qian, Peng Wang

**Affiliations:** 1 Department of Integrative Oncology, Fudan University Shanghai Cancer Center, 270 Dong An Road, Shanghai, 200032 China; 2 Department of Oncology, Shanghai Medical College, Fudan University, Shanghai, 200032 China

**Keywords:** *RAS* mutations, Hotspots, Clinicopathological features, RAS-targeted therapy

## Abstract

*RAS* mutations (*HRAS*, *NRAS*, and *KRAS*) are among the most common oncogenes, and around 19% of patients with cancer harbor *RAS* mutations. Cells harboring *RAS* mutations tend to undergo malignant transformation and exhibit malignant phenotypes. The mutational status of *RAS* correlates with the clinicopathological features of patients, such as mucinous type and poor differentiation, as well as response to anti-EGFR therapies in certain types of human cancers. Although RAS protein had been considered as a potential target for tumors with *RAS* mutations, it was once referred to as a undruggable target due to the consecutive failure in the discovery of RAS protein inhibitors. However, recent studies on the structure, signaling, and function of RAS have shed light on the development of RAS-targeting drugs, especially with the approval of Lumakras (sotorasib, AMG510) in treatment of KRAS^G12C^-mutant NSCLC patients. Therefore, here we fully review *RAS* mutations in human cancer and especially focus on emerging strategies that have been recently developed for RAS-targeting therapy.

## Background

*HRAS* was first regarded as oncogene due to a single-point mutation in 1982. Subsequently, *NRAS* and *KRAS* were identified quickly [1]. Since then, intense efforts have been made into the study of RAS [2]. Among the most common oncogenes regarding human cancers, mutant RAS affects approximately 19% of tumors [3].

RAS proteins belong to the family of GTPases and are considered as regulators of cellular proliferation, cell migration, apoptosis, and survival [2]. Mutant RAS proteins stimulate downstream signals and have significant oncogenic roles, and tumor cells harboring mutant RAS exhibit more aggressive phenotypes [4, 5]. Accordingly, tumor patients with mutant RAS possess a worse prognosis and shorter overall survival (OS) compared with those patients without RAS mutation [6, 7].

In clinical cancer patients, tumors harboring RAS mutations exhibit distinct clinicopathological characteristics and sensitivity to targeted therapy and chemotherapy. For example, RAS mutations are thought to correlate with features that predict aggressive behaviors, such as increased mitosis [8, 9]. RAS mutational status is also correlated with the efficiency of targeted therapy. For example, anti-EGFR therapy is unsuitable for RAS-mutant metastatic colorectal cancer (mCRC) patients [10–13]. However, whether the mutational status of RAS affects chemotherapy efficiency remains controversial.

Direct targeting of RAS proteins used to be considered impossible because of the lack of drug-binding pockets on the surface of RAS proteins. However, great efforts are being made to determine various targeting strategies including: (1) targeting upstream molecules (e.g., PDE δ, SHP2, and STK19); (2) targeting the RAS proteins directly (e.g., by chemical compound or antibody); (3) targeting the downstream effectors (e.g., RAF, MEK, ERK, PI3K, and combined inhibition); (4) RNA interference (RNAi) of *RAS* expression; (5) targeting the distinct metabolic processes correlated with RAS mutation (e.g., micropinocytosis and autophagy); and (6) screening for synthetic lethal interactors [14]. In certain types of cancers, an objective response has been observed, including for KRAS^G12C^ inhibitors AMG510 and MRTX849 in KRAS^G12C^-mutant lung or colorectal cancer patients, for the SRC homology-2-containing protein tyrosine phosphatase 2 (SHP2) inhibitor RMC-4630 in advanced NSCLC patients harboring KRAS mutation and for RAS/MEK inhibitor RO5126766 (VS-6766) combination with FAK inhibitor in KRAS mutation low-grade serous ovarian cancer (LGSOC) [15–17]. Remarkedly, based on a study of 124 advanced NSCLC patients harboring KRAS^G12C^ mutation, Lumakras (sotorasib, AMG510) were approved for KRAS^G12C^ NSCLC patients by the U.S. Food and Drug Administration (FDA) recently, which is the first approved targeted therapy for tumors with *KRAS* mutation [18, 19]. Therefore, here we fully review RAS mutations in human cancer and especially focus on emerging strategies that have been recently developed for RAS-targeted therapy.

## RAS structure, function, and signaling

**Fig. 1:**
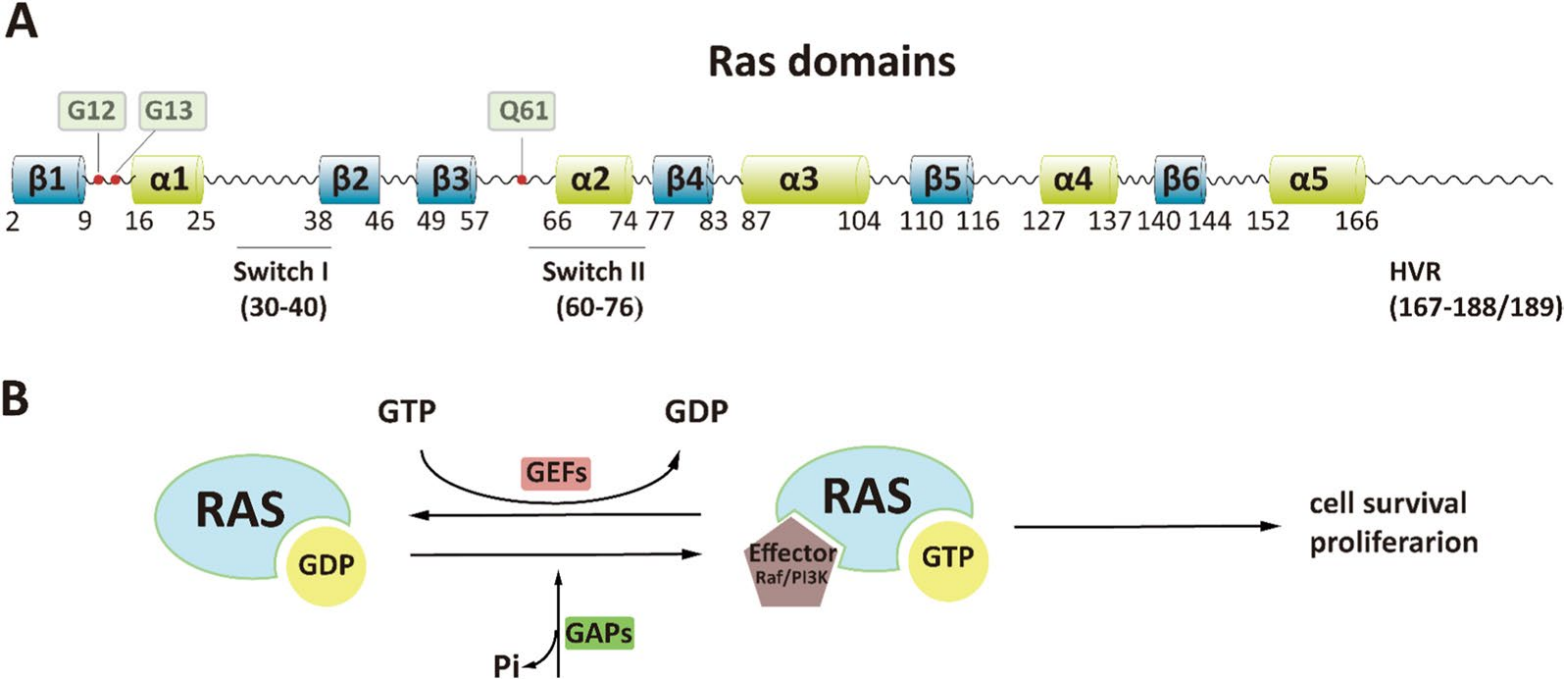
Structure and switch of RAS. **A** Structure of RAS proteins, including the effector lobe (aa 1–86), allosteric lobe (aa 87–165), and HVR (aa 167–188/189). Switch I (aa 30–40) and switch II (aa 60–76) are located in the effector lobe and function in effector binding and GEF or GAP binding. The HVR domain contributes to RAS binding to cell membranes. **B** Inactive GDP-bound KRAS and GTP-bound KRAS cycle. The switch to RAS-GTP is stimulated by GEF, while GAPs accelerate the termination of the active state. The active GTP-bound RAS transfers the proliferation and differentiation signals through downstream effectors such as RAF, PI3K, and RalGEFs

There are three *RAS* genes giving rise to four main protein products: KRAS4A, KRAS4B, NRAS, and HRAS. These isoforms share highly homogenous sequences or structures, and all possess conserved G domains (aa 1–166) and C-terminal hypervariable regions (HVRs) (aa 166–188/189) (Fig. 1A). The G domain of RAS, consisting of switch I (aa 30–40), switch II (aa 60–76), and a P loop (aa 10–17), is responsible for the binding of downstream effectors to transduce downstream signals, while the C-terminal has vital role in RAS binding to membranes [20]. The final four amino acids, CAAX, of the C-terminal are the targets of posttranslational modifications, including iso-prenylation, proteolysis, and methylation which mediate RAS shift and binding to the cell membrane [21].

RAS proteins cycle between the GDP-bound inactive state (RAS-GDP) and the GTP-bound active state (RAS-GTP) (Fig. 1B). The inactive state of RAS exchanges the GDP/GTP binding when signals provoke it, and the switch to RAS-GTP is accelerated by GEFs (e.g., SOS1). The rate of the intrinsic GTP hydrolysis of RAS proteins is very slow; As a result, GAPs accelerate the termination of the active state by several orders of magnitude [22]. The active RAS-GTP, interacting with downstream effectors including RAF, PI3K, and Ral guanine exchange factors (RalGEFs), transduces the signal to regulate biological behavior [23–26]. The first two corresponding pathways, RAS–RAF–MEK–ERK and RAS–PI3K-AKT–mTORC, act as fundamental signaling pathways of RAS proteins [24]. Mutations in the three RAS isoforms, G12, G13, and Q61, can abolish the intrinsic GTPase activity of RAS and increase the GEF-mediated exchange rate [27–29]. As a result, RAS remains a continuously active GTP-bound state and hence is oncogenic.

## RAS mutation frequency and hotspots in human cancers

**Fig. 2:**
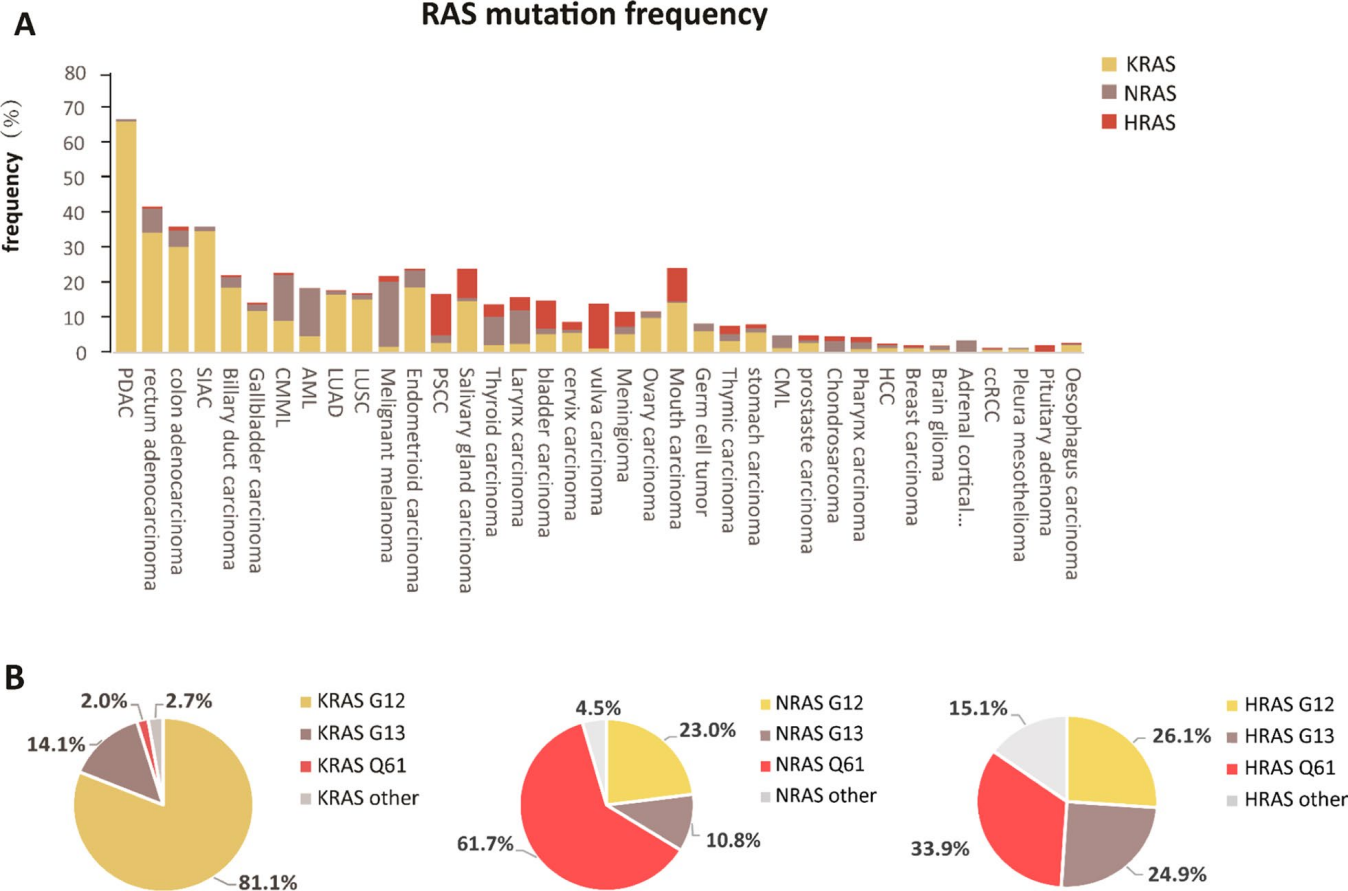
RAS mutational frequency and hotspots in human cancer. **A** The mutational frequency of KRAS, NRAS, and HRAS in various cancers. **B** The proportion of RAS mutation hotspots. G12, G13, and Q61 occupy 96–98% of all mutations in KRAS and NRAS isoforms. The data are taken from the Catalogue of Somatic Mutations in Cancer (COSMIC v94). PDAC, pancreatic ductal adenocarcinoma; LUAD, lung adenocarcinoma; CMML, chronic myelomonocytic leukemia; AML, acute myeloid leukemia; LUSC, lung squamous cell carcinoma; CML, chronic myelogenous leukemia; HCC, hepatocellular carcinoma; ccRCC, clear cell renal cell carcinoma

RAS mutations occur in approximately 19% of all cancers, occupying a prominent role in tumorigenesis and tumor progression [3]. Among which, KRAS is the most frequently mutated isoform, followed by NRAS, and HRAS. RAS isoform mutations show selectivity in various human cancers (Fig. 2A). For example, KRAS mutations often occur in pancreatic duct adenocarcinoma (PDAC), lung adenocarcinoma (LUAC), and colon and rectal adenocarcinoma (with mutation frequencies of 66.1%, 16.5%, 30.3%, and 34.4%, respectively; COSMIC v94), whereas in hematological malignancies such as chronic myelomonocytic leukemia (CMML) and acute myeloid leukemia (AML), *NRAS* mutation frequencies are relatively high at up to 13.1% and 13.6% (COSMIC v94), respectively, reflecting the rates in malignant melanoma, thyroid carcinoma, and larynx carcinoma, which have NRAS mutation frequencies of 18.6%, 8.1%, and 9.7%, respectively (COSMIC v94). Although HRAS mutations are negligible in human cancers, salivary gland carcinoma, mouth carcinoma, and vulva carcinoma possess relatively high rates of HRAS mutation.

Although > 100 mutation sites have been identified in all three RAS isoforms, the most prominent mutational hotspots are G12, G13, and Q61, occupying almost 96%–98% of all mutations in KRAS and NRAS isoforms, whereas the proportion of HRAS mutations is relatively low (COSMIC v94). Furthermore, different RAS isoforms exhibit varied hotspot preference for G12, G13, and Q61. Approximately 80% of mutations reside at G12 for KRAS mutations; in contrast, Q61 mutations are more common in NRAS, accounting for 60% of all mutations. Regarding HRAS, the mutational frequency among the three sites is similar (Fig. 2B). The underlying mechanism of codon-specific RAS mutations in specific tumor types remains unclear. Recent observations indicate that codon-specific mutations that confer a fitness advantage to tumor cells may explain the selection. For example, mouse models of knocked-in NRAS^Q61R^ exhibited melanoma formation, but those with NRAS^G12D^ did not; the mechanism lied in increased GTP binding affinity and reduced intrinsic GTPase activity compared with NRAS^G12D^ [30]. In fact, the isoform, codon, and frequency of RAS mutation vary by tissue type.

## Clinical implications of RAS mutations

### Correlation of RAS mutations and clinicopathological features

RAS mutational status correlates with clinicopathological features. Patients harboring mutant RAS exhibit distinct phenotypes, clinical pathology classification, and staging (Table 1). RAS mutations represent aggressive biological behavior of thyroid cancer and colorectal cancer [31]. As a result, colorectal cancer patients harboring KRAS or NRAS have shorter overall survival (OS) [32]. Additionally, KRAS status will shift the metastatic profile of colorectal cancer; KRAS-mutant tumors tend to spread to the lungs, whilst wild-type tumors have a higher propensity of invasion to the liver [33, 34]. For patients with colorectal liver metastases receiving liver resection, studies have indicated that KRAS mutations correlate with worse recurrence-free survival (RFS) and OS [35–38]. In melanoma, NRAS mutations correlate with the presence of mitoses, lower tumor-infiltrating lymphocyte (TIL) grade, extremity location, thick tumors, and higher AJCC stage [8, 9, 39]. In ovarian cancer, significant associations are found between KRAS mutations and lower grade, mucinous histological subtype, and positive progesterone expression [40]. These findings suggest that patients with RAS mutations possess distinct clinicopathological features.

**Table 1:** Clinicopathological features of patients with RAS mutations

Tumor type	N	RAS mutation	Mutation rate	Mutation site	Clinicopathologic features	References
Melanoma	912	NRAS	13.0%	Codon 12, 13, 61	Presence of mitoses; lower TIL grade; anatomic site other than scalp/necks	[39]
Thyroid cancer	107	HRAS, NRAS, KRAS	32.7%	NM	Poorly or undifferentiated type;	[31]
mCRC	484	KRAS, NRAS	51.6%	Codon 12, 13, 61, 146	More mucinous type; higher lung metastases tendency; right-side preference of primary tumors	[206]
CRC	926	KRAS	14.7%	Codon 12, 13	Villous histology preference; advanced adenomas; older age	[207]
NSCLC	6583	KRAS	9.2%	Codon 12, 13	More mucinous type; frequent poorly-differentiated grade; solid pattern tumors preference; larger sized tumors	[208]
IMA	45	KRAS	48.9%	Codon 12	Located in the lower lung lobe; lower frequency of nuclear atypia; lower proportion of geminin-positive cell	[209]
EOC	153	KRAS	11.1%	Codon 12, 13, 61	More mucinous type; lower differentiation grade; higher PR expression; higher pT classifications	[40]
SIA	190	KRAS	32.1%	Codon 12, 13	More frequent pancreatic invasion	[210]

EOC epithelial ovarian cancer, SIA small intestinal adenocarcinoma, IMA invasive mucinous adenocarcinoma of the lung, CRC colorectal cancer, mCRC metastatic colorectal cancer, PR progesterone receptor, TIL tumor-infiltrating lymphocytes, NM not mentioned

### Correlations of RAS mutational status and treatment efficiency of targeted therapy, chemotherapy, and/or immunotherapy

Recent studies have identified correlations between RAS mutational status and the treatment efficiency of targeted therapy, chemotherapy, and immunotherapy (Table 2). KRAS mutational status predicts the response to anti-epidermal growth factor receptor (anti-EGFR) therapy in patients with mCRC. Patients with KRAS mutations in exons 2 do not benefit from anti-EGFR therapy; either anti-EGFR antibody alone or combined with chemotherapy [10–12, 41]. However, there seems to be an exception for patients with KRAS^G13D^, who benefit from cetuximab [42, 43]. In contrast with the clear predicted significance of KRAS mutational status for anti-EGFR therapy in mCRC, whether it can be utilized in NSCLC remains controversial. An association between KRAS mutations and lack of response to anti-EGFR has been observed in the clinic [44, 45]; thus, it is reasonable to hypothesize that NSCLC tumors with KRAS mutations are resistant to anti-EGFR therapy. However, increased studies have indicated that KRAS mutational status does not have predictive significance in the selection of patients for anti-EGFR therapy in NSCLC [46–49]. Therefore, KRAS mutational status currently provides insufficient evidence to recommend the selection of patients for anti-EGFR treatment in NSCLC.

**Table 2:** Clinical trials in terms of the predictive value of RAS mutational status to treatment response

Study	No. Total	No. Mut	Tumor stage	Treat	OS (mon)	HR (95%CI)	P	PFS (mon)	HR (95%CI)	P	References
*Targeted therapy*											
Lievre, A., et al	89	24	mCRC	Cetuximab for KMP vs KWP	10.1 vs 14.3	ND	.026	2.4 vs 7.3	ND	**.**0001	[11]
NCT00079066	572	164	mCRC	Cetuximab + BSC vs BSC For KMP	4.5 vs 4.6	0.98 (0.70–1.37)	.89	1.8 vs 1.8	0.99 (0.73–1.35)	.96	[211]
Amado, R. G., et al	427	184	mCRC	Panitumumab + BSC vs BSC For KMP	4.9 vs 4.4	1.02 (0.75–1.39)	ND	1.7 vs 1.7	0.99 (0.73–1.36)	ND	[212]
OPUS	337	99	mCRC	Cetuximab + FOLFOX4 vs FOLFOX4 for KMP	NA	ND	ND	5.5 vs 8.6	1.83 (1.10–3.05)	.0192	[213]
NCT00154102	1198	397	mCRC	Cetuximab + FOLFIRI vs FOLFIRI for KMP	16.2 vs 16.7	1.035 (0.834–1.284)	.75	7.4 vs 7.7	1.17 (.887–1.544)	.26	[214]
NCT00364013	1096	440	mCRC	Panitumumab + FOLFOX4 vs FOLFOX4 for KMP and NMP	15.6 vs 19.2	1.25 (1.02–1.55)	.03	7.3 vs 8	1.31 (1.07–1.60)	ND	[41]
NCT00339183	1083	486	mCRC	Panitumumab + FOLFIRI vs FOLFIRI for KMP	11.8 vs 11.1	ND	ND	5.0 vs 4.9	ND	.14	[215]
NCT00145314	1064	195	mCRCIV	Cetuximab + FLOX vs FLOX KMP for KMP	21.1 vs 20.4	1.03 (0.68–1.57)	.89	9.2 vs 7.8	0.71 (0.50–1.03)	.07	[216]
NCT01000025	720	78	NSCLC IV	Dacomitinib vs placebo for KMP	5.82 vs 8.28	2.10 (1.05–4.22)	NA	1.61 vs 1.86	1.34 (0.78–2.29)	ND	[217]
NCT00637910	219	51	NSCLC IV	Docetaxel vs Erlotinib for KMP	NA	0.81 (0.45–1.47)	ND	NA	0.89 (0.51–1.57)	ND	[218, 219]
TRIBUTE	274	55	NSCLC IIIB or IV	Erlotinib + CP vs CP for KMP	4.4 vs 13.5	NA	.019	NA	ND	ND	[44]
TRUST	311	17	NSCLC IIIB or IV	Erlotinib for KMP vs KWP	NA	1.64 (0.97–2.80)	.064	NA	1.56 (0.92–2.65)	.094	[220]
NCIC CTG PA.3	569	92	aPC	Erlotinib + Gem vs placebo + Gem for KMP	6.0 vs 7.4	1.07 (0.68–1.66)	.78	NA	ND	ND	[221, 222]
NCT00440167	281	121	aPC	Gem + Erlotinib/Cap or Cap + Erlotinib/Gem for KWP vs KMP	7.9 vs 5.7	1.68 (1.17–2.41)	.005	NA	ND	ND	[223]
NCT01267344	122	44	aBTC	Gem, Ox + Cetuximab vs Gem, Ox For KMP	NA	0.73 (0.39–1.35)	.313	NA	ND	ND	[224]
*Chemotherapy*											
CALGB 89803	508	178	CRC III	FU/LV or IFL for KMP vs KWP	NA	0.86 (0.60–1.23)	ND	NA	0.95 (0.70–1.28)	ND	[225]
TRIBUTE	274	55	NSCLC IIIB or IV	CP for KMP vs KWP	13.5 vs 11.3	ND	ND	NA	ND	ND	[44]
OPUS	337	99	mCRC	FOLFOX4 for KMP vs KWP	NA	ND	ND	8.6 vs 7.2	1.404 (0.867–2.271)	ND	[213]

KMP KRAS-mutant patients, NMP NRAS-mutant patients, KWP wild-type KRAS patients, BSC best supportive care, mCRC metastatic colorectal cancer, NSCLC non-small-cell lung carcinoma, aPC advanced pancreatic cancer, aBTC advanced biliary tract cancer, FOLFOX4 oxaliplatin, fluorouracil, and leucovorin, FOLFIRI 5-fluorouracil, folinic acid, and irinotecan, Gem gemcitabine, CP carboplatin and paclitaxel, Cap capecitabine, Ox oxaliplatin, IFL fluorouracil [5-FU], leucovorin, and irinotecan, FU/LV 5FU + leucovorin, NA none, ND not determined

Whether RAS mutational status influences chemotherapy efficiency remains unclear, and the predictive value of mutant RAS status to the response to chemotherapy is controversial [50–54]. Improved clinical response to chemotherapy was observed in KRAS-mutant patients suffering from mCRC and pancreatic neuroendocrine neoplasm grade-3 (PanNEN-G3) [54–56], while in other clinical trials, KRAS mutational status did not have prognostic value for stage II/III colon cancer receiving either FU/FA alone or in combination with irinotecan [52]. Further analyses from the PETACC-8 trial even suggested that KRAS mutation was associated with shorter DFS and OS for stage III colon cancer treated with leucovorin, fluorouracil, and oxaliplatin alone or combination with cetuximab, while in patients with microsatellite instability (MSI), KRAS mutational status did not have prognostic value [50, 53].

With the emergence of drugs targeting negative immune regulators containing programmed cell death protein 1(PD1), programmed cell death 1 ligand 1(PD-L1), or cytotoxic lymphocyte antigen 4 (CTLA-4), immune therapy represented by immune checkpoint blockade (ICB) has revolutionized cancer treatment [57]. It has been found that high PD-L1 expression was significantly correlated with the presence of KRAS mutations in pulmonary sarcomatous carcinoma and lung adenocarcinoma [58, 59], indicating that patients with KRAS mutations may exhibit a more efficient response to ICB. In addition, ICB tended to show consistently higher efficiency in KRAS-mutant NSCLC [60]. However, oncogenic KRAS promotes tumor cell immune escape and immune therapy resistance through attracting immune-suppressive cells or suppressing cytotoxic cells in a colorectal cancer mouse model [61, 62]. Therefore, whether RAS mutational status should be considered before administering ICB therapy warrants further study.

## RAS targeting strategies

**Fig. 3:**
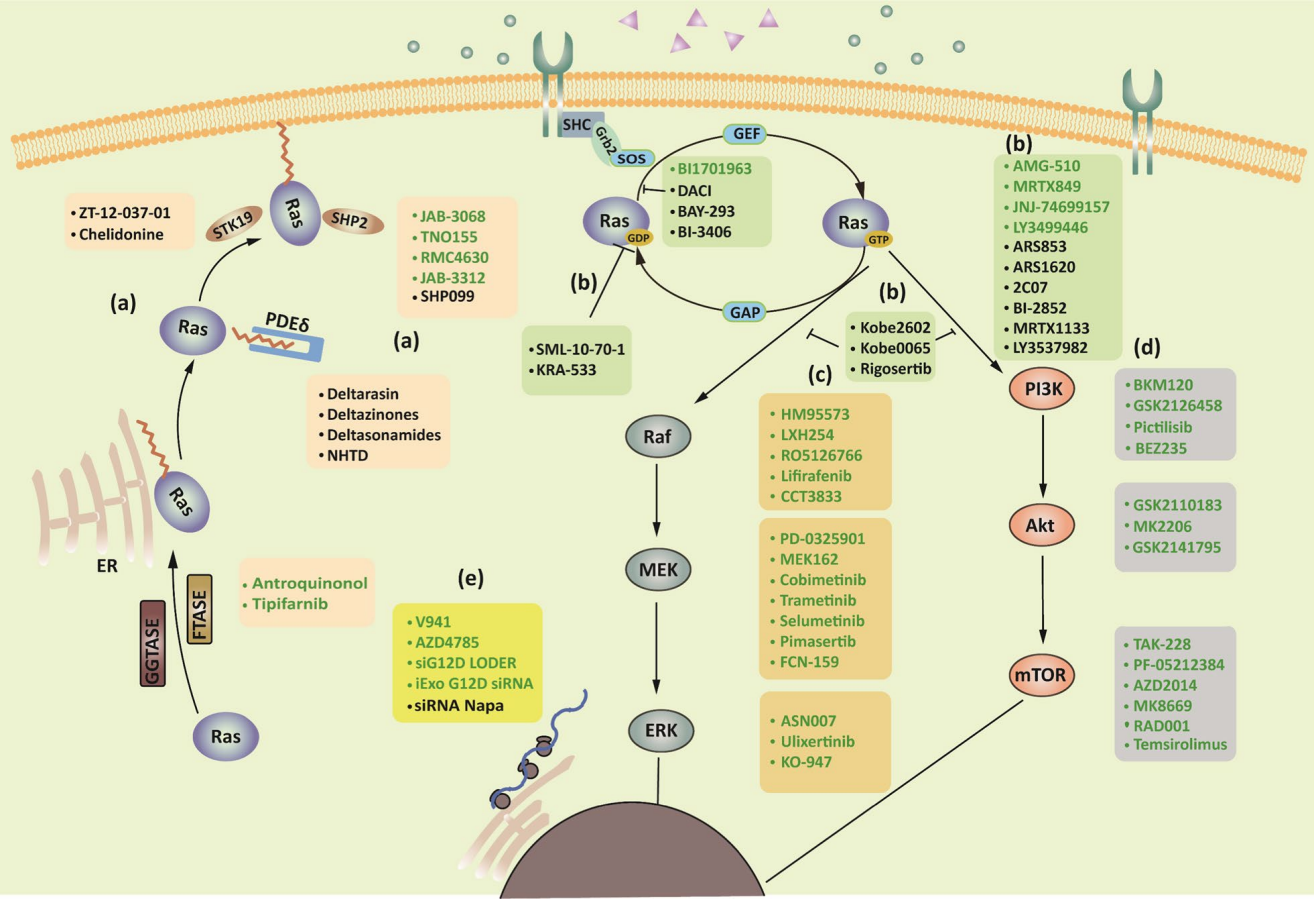
Preclinical and clinical drugs targeting RAS-mutant tumors. (a) Targeting upstream molecules of RAS. Promising targets include PDEδ, SHP2, and STK19. (b) Targeting RAS directly, especially G12C covalent binders. (c) Targeting downstream RAF–MEK–ERK signaling. (d) Targeting downstream PI3K–AKT–mTOR signaling. (e) RNA interference of mutant *RAS* mRNA. Molecules that underwent clinical trials are indicated in green, those that are now under preclinical evaluation are indicated in lack. Napa, nanoparticle

Drugging RAS proteins directly used to be considered impossible because of the lack of pockets for drug binding on the surface of RAS proteins, and hence, the focus shifted to upstream and downstream proteins of RAS with the aim of suppressing the oncogenic signal. Recent studies on RAS structure, function, and signaling have revealed new insights on the development of RAS targeting strategies. Targeting upstream proteins, downstream proteins, and RAS directly, as well as RNA interference, represent the direct suppression of RAS oncogenic signals. Preclinical or clinical drugs that directly disturb RAS oncogenic signaling are shown in Fig. 3. In addition, RAS mutations bring specific characteristics, such as distinct metabolic processes and antigens, revealing indirect strategies that make targeting these characteristics feasible [63, 64]. Here, we summarize promising drugs with direct and indirect strategies in preclinical or clinical development (Tables 3 and 4).

**Table 3:** Potential molecules targeting RAS directly in preclinical or clinical trials

Target RAS directly						
DACI	HEK-293 T	KRAS Mut	Pre	NA	Inhibit SOS-Ras interaction	[88]
BAY-293	NSCLC cell lines	KRAS G12C	Pre	NA	Inhibit SOS-Ras interaction	[90]
BI 3406	Cell lines	KRAS Mut lnc	Pre	NA	Inhibit SOS-Ras interaction	[226]
BI 1701963	Solid tumors	KRAS Mut	Clinical I	Recruiting	Inhibit SOS-Ras interaction Single agent or comb Trametinib	NCT04111458
SML-10-70-1	NSCLC cell lines	KRAS G12C	Pre	NA	Inhibit GN binding	[101, 102]
KRA-533	NSCLC xenografts	KRAS K117A	Pre	NA	Inhibit GN binding	[103]
Rigosertib	PanIN; CRC NSCLC xenograft models	KRAS G12D G13D G12S	Pre	NA	Inhibit Ras effectors interaction	[94]
Kobe2602 Kobe0065	CRC xenograft models	KRAS G12V	Pre	NA	Inhibit Ras effectors interaction	[104]
ARS853	NSCLC cell lines	KRAS G12C	Pre	NA	Target inactive Ras	[91, 92]
ARS1620	NSCLC xenograft models	KRAS G12C	Pre	NA	Target inactive Ras	[93]
LY3537982	NSCLC PDX	KRAS G12C	Pre	NA	Target inactive Ras	[227]
MRTX1133	PDAC xenograft	KRAS G12D	Pre	NA	Target inactive Ras	[100]
2C07	NA	HRAS M72C	Pre	NA	Target inactive Ras	[96]
BI-2852	NCI-H358 cell	KRAS G12D	Pre	NA	Target surface pocket of RAS	[97]
AMG510	Mut solid tumors	KRAS G12C	Clinical I/II	Recruiting	Target inactive Ras Single agent	NCT03600883() (CodeBreaK 100)
	Mut solid tumors	KRAS G12C	Clinical I	Recruiting	Target inactive Ras Single agent	NCT04380753 (CodeBreaK105)
	NSCLC	KRAS G12C	Clinical III	Not yet recruiting	Target inactive Ras Compare with Docetaxel	NCT04303780 (CodeBreaK200)
	Advanced solid tumors	KRAS G12C	Clinical Ib/II	Recruiting	Targeting inactive Ras Comb with MEKi, PD1i, PDL1i, SHP2i Pan-ErbBi, EGFRi + chemotherapy	NCT04185883 (CodeBreaK101)
MRTX849	Advanced solid tumors	KRAS G12C	Clinical I/II	Recruiting	Target inactive Ras Single agent and comb With Pembrolizumab/Cetuximab/Afatinib	NCT03785249
	Advanced solid tumors	KRAS G12C	Clinical I/II	Recruiting	Comb with TNO155	NCT04330664
LY3499446	Solid tumors NSCLC, CRC	KRAS G12C	Clinical I/II	Terminated	Target inactive Ras Single agent or comb Single agent or comb with Abemaciclib/Cetuximab Erlotinib/Docetaxel	NCT04165031
JNJ-74699157	Solid tumors NSCLC, CRC Neoplasms	KRAS G12C	Clinical I	Completed	Target inactive Ras	NCT04006301

mCRC metastatic colorectal cancer, NSCLC non-small-cell lung carcinoma, PanIN pancreatic intra-epithelial neoplasia, PDAC pancreatic ductal adenocarcinoma, Mut mutation, lnc include, means including patients with RAS mutation, NA, none

**Table 4:** Potential molecules targeting RAS signaling in preclinical or clinical trials

Targets	Molecule	Tumor Type/model	RAS Mut	Phase	Status	Notes	Referencess
*Target the upstream*							
FTase/GGTase	Antroquinonol	PDAC	KRAS mut lnc	Clinical I/II	Recruiting	Single agent	NCT03310632
		NSCLC	NM	Clinical I	Completed	Single agent	NCT01134016
	tipifarnib	TC HNSCC SCC	HRAS	Clinical II	Completed	Single agent	NCT02383927 [69]
		UC	HRAS	Clinical II	NM	Single agent	[70]
PDEδ	Deltarasin	Xenografted PDAC modelss	KRAS	Pre	NA	Single agent	[75]
	Deltazinones	PDAC cell lines	KRAS	Pre	NA	Single agent	[77]
	Deltasonamides	PDAC cell lines	KRAS	Pre	NA	Single agent	[228]
	NHTD	Xenografted NSCLC models	KRAS	Pre	NA	Single agent	[79]
STK19	ZT-12–037-01 (1a)	Melanoma xenograft models	NRAS	Pre	NA	Single agent	[86]
	Chelidonine	Melanoma xenograft models, cell lines	NRAS	Pre	NA	Single agent	[87]
SHP2	SHP099	PDAC, NSCLC xenograft models	KRAS	Pre	NA	Single agent	[84]
	JAB-3068	NSCLC, HNC, ESC, Other solid tumors	NM	Clinical I/II	Recruiting	Single agent	NCT03565003
	JAB-3312	NSCLC, CRC, PDAC, BC, ESC	KRAS G12 mut lnc	Clinical I	Recruiting	Single agent	NCT04045496
	TNO155	NSCLC, CRC	KRAS G12C Mut lnc	Clinical I	Recruiting	Single agent	NCT03114319
	RMC-4630	PC, OVCA, OEC, ESC, NF1	KRAS G12 Mut lnc	Clinical I	Recruiting	Single agent	NCT03634982
		Solid tumors	KRAS mut lnc	Clinical Ib/II	Recruiting	Comb with Cobimetinib	NCT03989115
*Target the downstream*							
RAF	HM95573	Solid tumors	KRAS, NRAS	Clinical I	Completed	Single agent	NCT03118817
	RO5126766(VS-6766)	NSCLC	KRAS	Clinical I	Active, not recruiting	Dual MEK/Raf inhibitor	NCT03681483
		NSCLC	KRAS	Clinical I	Recruiting	Comb with FAK inhibitor (VS-6063)	NCT03875820
		LGSOC	KRAS	Clinical II	Recruiting	Comb with FAK inhibitor (VS-6063)	NCT04625270
	LXH254	NSCLC, MM	KRAS, NRAS	Clinical Ib	Recruiting	Comb with MEK, ERK, or CD4/6 inhibitors	NCT02974725
	Lifirafenib	MM, TC, OC, NSCLC, CRC, EC	KRAS, NRAS	Clinical I	completed	Single agent	[131]
	CCT3833	MM	RAS Mut lnc	Clinical I	completed	Single agent	NCT02437227
MEK1/2	PD-0325901	NSCLC	KRAS	Clinical I/II	Active, not recruiting	Comb with CD4/6 inhibitor (Palbociclib)	NCT02022982
		NSCLC	KRAS	Clinical I/II	Recruiting	Comb with pan-HER inhibitor (Dacomitinib)	NCT02039336
		NSCLC, EC, CRC, OC, TC, PC, MM	KRAS	Clinical I/II	Recruiting	Comb with dual BRAF and EGFR, Inhibitor (BGB-283)	NCT03905148
	MEK162	NSCLC	KRAS	Clinical I/II	Recruiting	Comb with CDK4/6 inhibitor (Palbocicib)	NCT03170206
		NSCLC	KRAS	Clinical I/Ib	Active, not recruiting	Comb with EGFR inhibitor (Erlotinib)	NCT01859026
		PC CRC NSCLC MM	KRAS NRAS Mut lnc	Clinical I	Completed	Comb with PI3K inhibitor (BKM120)	NCT01363232
		Solid tumors	KRAS NRAS Mut lnc	Clinical I	Completed	comb with AKT inhibitor (BEZ235)	NCT01337765
		PC, NSCLC	KRAS NRAS	Clinical Ib/II	Terminated	Comb with PARP and PDL1 inhibitor (Talazoparib, Avelumab)	NCT03637491
	Cobimetinib	NSCLC, CRC	KRAS	Clinical I	Completed	Comb with MEHD7945A	NCT01986166
	Trametinib	NSCLC	KRAS, NRAS Mut lnc	Clinical II	Completed	Single agent	NCT01362296
		mCRC	KRAS Mut lnc	Clinical Ib/II	Terminated	Comb with CDK4/6 inhibitor (ribociclib)	NCT02703571
		NSCLC, PC	KRAS	Clinical Ib/II	Recruiting	Comb with Bcl-2 inhibitor (Navitoclax)	NCT02079740
		Multiple Myeloma	KRAS NRAS Mut lnc	Clinical I	Recruiting	Comb with BRAF inhibitor (Dabrafenib)	NCT03091257
		NSCLC	KRAS Mut lnc	Clinical I/II	Active, not recruiting	Comb with PD1 inhibitor (Pembrolizumab)	NCT03225664
		MM	NRAS	Clinical Ib/II I/II	Terminated	Comb with ERBB3 inhibitor (CDX-3379)	NCT03580382
		Solid tumors DTC	NRAS lnc	Clinical I	NA	Comb with VEGF inhibitor (Pazopanib)	[13]
	Selumetinib	NSCLC	KRAS	Clinical II	Withdrawn	Comb with PDL1 inhibitor (durvalumab)	NCT03004105
		NSCLC	KRAS	Clinical I/II	Recruiting	Comb with EGFR inhibitor (Afatinib)	NCT02450656
		mCRC	KRAS Mut lnc	Clinical II	Completed	Comb with chemotherapy (Irinotecan)	NCT01116271
		NSCLC	KRAS Mut lnc	Clinical Ib/II	Active, not recruiting	Comb with mTOR inhibitor (AZD2014)	NCT02583542
		mCRC	KRAS	Clinical II	Completed	Comb with AKT inhibitor (MK-2206)	NCT01333475
		NSCLC	KRAS	Clinical III	Active, not recruitingrecruiting	Comb with chemotherapy (Docetaxel)	NCT01933932
	Pimasertib	MM	NRAS	Clinical II	Completed	Single agent	NCT01693068
	FCN-159	MM	NRAS	Clinical I	Recruiting	Single agent	NCT03932253
ERK1/2	ASN007	MM, CRC, NSCLC	KRAS, NRAS Mut lnc	Clinical I	Completed	Single agent	NCT03415126
	Ulixertinib	Solid tumors	KRAS, NRAS HRAS Mut lnc	Clinical II	Suspended	Single agent	NCT03698994
	KO-947	Solid tumors	KRAS, NRAS HRAS Mut lnc	Clinical I	Terminated	Single agent	NCT03051035
	SCH772984	Pancreatic	KRAS	Pre	NA	Single agent	[154]
		Xenograft Models					
	AZD0364	NSCLC CRC Xenograft Models	KRAS	Pre	NA	Comb with MEK inhibitor (selumetinib)	[157]
PI3K	PF-05212384	NSCLC	KRAS Mut	Clinical I	Terminated	Comb with MEK inhibitor (PD-0325901)	NCT01347866
	BKM120	Solid tumors	KRAS Mut lnc	Clinical I	Completed	Comb with MEK inhibitor (GSK1120212)	NCT01155453
	GSK2126458	Solid tumors	KRAS Mut lnc	Clinical I	Terminated	Comb with MEK inhibitor (GSK1120212)	NCT01248858
	Pictilisib	Solid tumors	KRAS Mut lnc	Clinical I	Terminated	Comb with MEK inhibitor (cobimetinib)	NCT00996892
Akt	MK2206	NSCLC	KRAS	Clinical I	Completed	Comb with MEK inhibitor (AZD6244)	NCT01021748
		CRC	KRAS	Clinical II	Completed	Comb with MEK inhibitor (AZD6244)	NCT01333475
	GSK2141795	AML	KRAS/NRAS Mut	Clinical II	Terminated	Comb with MEK inhibitor (Trametinib)	NCT01907815
	GSK2110183	Solid tumors	KRAS Mut lnc	Clinical I	Completed	Comb with MEK inhibitor (Trametinib)	NCT01476137
mTOR	TAK-228	SCLC	KRAS	Clinical II	Completed	Single agent	NCT02417701
	MK8669	NSCLC	KRAS	Clinical II	Terminated	Single agent	NCT00818675
	Temsirolimus	mCRC	KRAS	Clinical II	Completed	Comb with chemotherapy (Irinotecan)	NCT00827684
	Everolimus	mCRC	KRAS	Clinical II	Completed	Single agent	NCT00419159
		NSCLC	KRAS	Clinical I	Completed	Comb with Sorafenib	NCT00933777
		EC	KRAS	Clinical II	Completed	Single agent	NCT00870337 [184]
*RNA interference*							
	siRNA-Loaded nanoparticles	NSCLC cell lines	KRAS	Pre	NA	Single agent	[114]
	AZD4785	NSCLC mCRC	KRAS	Clinical I	Completed	Single agent	NCT03101839
	siG12D LODERAZD4785	LAPCNSCLC, mCRC	KRAS G12D	Clinical I	Completed	Single agent	[120]
	iExosomes G12D	PDAC	KRAS G12D	Clinical I	Recruiting	Single agent	NCT03608631
	siRNA						
	V941	Advanced PDAC, CRC, NSCLC	KRAS Mut	Clinical I	Recruiting	Single agent or Comb with Pembrolizumab	NCT03948763
*Target metabolic process*							
	Chloroquine	PDAC, MM xenograft models	KRAS, NRAS	Pre	NA	Comb with MEK inhibitor (Trametinib)	[191]
		PDAC xenograft	KRAS	Pre	NA	Comb with ERK inhibitor	[190]
		Models				(SCH772984)	
*Other strategies*							
	Anti-KRAS	PC, GC, RC, GICA	KRAS G12D	Clinical I/II	Suspended	Single agent	NCT03190941
	G12 mTCR		G12V Mut				NCT03745326
	PBL						
	CRISPR/Cas9	NSCLC xenograft modelsMODEL	KRAS G12S	Pre	NA	Single agent	[118]
	System	Model					
	PROTACs	NSCLC cell lines	KRAS G12C	Pre	NA	Single agent	[194, 195]
		NIH-3T3	KRAS G12V	Pre	NA	dTAG system	[198]
		NIH-3T3	KRAS G12V	Pre	NA	HaloPROTACs	[197]
		A549	HRAS	Pre	NA	HaloPROTAC comb with	[199]
			KRAS G12S			L-AdPROM	
		SW480	KRAS	Pre	NA	PROTAC (PDEδ)	[200]
		NSCLC cell lines	KRAS mut	Pre	NA	PROTAC (TBK1)	[201]

mCRC metastatic colorectal cancer, NSCLC non-small-cell lung carcinoma, PC pancreatic cancer, OVCA ovarian cancer; OEC, ovarian epithelial cancer, ESC esophageal carcinoma, NF1 neurofibromatosis type 1, GC gastric cancer, CC colon cancer, RC rectal cancer, UC urothelial carcinoma, GICA gastrointestinal cancer, EC endometrial cancer, PanIN pancreatic intra-epithelial neoplasia, LAPC locally advanced pancreatic cancer, MM malignant melanoma, PBL peripheral blood lymphocytes, Mut mutation, lnc include, means including patients with RAS mutation, NA, none. PROTACs PROteolysis TArgeting Chimeras

### Targeting upstream proteins

RAS proteins shift to the membrane for their biological activity. As a result, the idea of disrupting RAS translocation to cell membrane was proposed, especially in confronting the difficult approach of direct targeting of RAS. The initial attempt was to drug farnesyltransferase (FTase), which modifies the CAAX motif of RAS by farnesyl moiety addition. However, FTase inhibitors (FTIs) exhibited disappointing results in clinical trials toward to pancreatic cancer, which mainly possess KRAS mutation [65–67], and subsequent research revealed KRAS and NRAS gained alternative modifications by geranylgeranyltransferases (GGTase) in cells treated with FTIs [68]. Noteworthily, tipifarnib, a FTase inhibitor, exhibited encouraging efficiency in cancer harboring HRAS mutation [69, 70]. Although simultaneous inactivation of FTase and GGTase exhibited tumorigenesis inhibition in mouse models [71, 72], the toxicity associated with GGTIs limited their utility, thus reducing the benefit of targeting KRAS through combined FTase and GGTase inhibition [73]. Remarkedly, a bioactive natural compound from antrodia camphorata, antroquinonol, suppressed the proliferation of tumor cells in vitro and in vivo. The potential mechanism was the inhibition of RAS through inactivation of FTase and GGTase [74].

Recently, another target, phenyl-binding protein phosphodiesterase δ (PDEδ), has attracted attention. PDEδ facilitates RAS protein transport to either the endosomes or the Golgi, from where RAS shifts to the plasma membrane. It was found to disrupt the interaction between KRAS–PDEδ to suppresses KRAS signaling, thus impairing the proliferation of PDAC cells in vitro and in vivo [75, 76]. The three molecules, NHTD, deltarasin, and deltazinone, competitively bind the prenyl-binding pocket of PDEδ, exhibiting the ability to impair the RAS protein stimulation at the membrane and further suppressing oncogenic KRAS signaling [77–79]. However, additional clinical study is required to test their toxicity and efficiency in patients.

In addition to interfering with the plasma localization of RAS proteins, targeting kinases or phosphatases that regulate RAS activity also represent an alternative option. Tyrosine-protein phosphatase non-receptor type 11 (PTPN11), also known as SHP2, is a mediator associated the stimulation of the downstream RAS–RAF–MEK–ERK pathway, promoting MAPK signal activation [80]. Although SHP099, an SHP2 inhibitor, displayed minimal anti-proliferation effects in KRAS or BRAF mutant cell lines in vitro, it shrank KRAS-mutant tumors in vivo [81, 82]. In addition, combined inhibition of MEK and SHP2 showed high efficiency in engineered or xenograft KRAS-mutant pancreas, ovarian, and lung cancer [81, 83–85], overcoming the rapid resistance to MEK inhibitor as a single therapy. Recently, the SHP2 inhibitor RMC-4630 exhibited an encouraging disease control rate (DCR) of 67% for advanced NSCLC patients harboring KRAS mutations [16]. The novel SHP2 inhibitors JAB-3068 and JAB-3312 are also under clinical investigation for safety and preliminary antitumor activity in KRAS-mutant solid tumors (NCT03565003 and NCT04045496, respectively).

The serine/threonine kinase STK19 was recently identified as another NRAS activator. STK19 phosphorylates NRAS protein at serine 89 and improved NRAS binding to its effectors. Consequently, STK19 inhibitor ZT-12-037-01 (1a) could inhibit oncogenic NRAS-mediated melanoma growth in vitro and in vivo [86]. Recently, we screened out a new pharmacological inhibitor of STK19 named chelidonine, which could suppress the growth of NRAS-mutant tumors in vitro and in vivo [87].

### Direct targeting of RAS

Drugging RAS proteins directly used to be considered impossible. Although the guanine nucleotide (GN) binding site seems an ideal pocket, the sub-nanomolar affinity of GDP and GTP binding to RAS and their low intercellular concentrations make competitive nucleotide binding challenging. However, in recent years, targeting of RAS proteins directly has had a resurgence because of new findings in its crystal structure. The strategies include inhibiting the SOS–RAS interaction, trapping RAS in its inactive conformation, targeting the GN binding site, and hindering RAS effector interaction. Remarkedly, the KRAS^G12C^ inhibition acquired great breakthrough, especially with the recent approval of AMG510 for KRAS^G12C^ NSCLC patients, the history of undruggable target of RAS in the clinic ended.

First, inhibition of SOS-mediated nucleotide exchange activity was shown to make sense [88, 89]. DACI, a small molecule identified in a fragment screen, was found to bind the pocket of the RAS–SOS interaction surface. DACI restrained nucleotide exchange by blocking the interaction of RAS and SOS and inhibiting RAS activation in transformed cells [88]. Furthermore, another compound, BAY-293, selectively suppresses KRAS–SOS interaction with a proper IC50, is thus a promising compound for further investigation [90]. Remarkedly, another compound that disturbs RAS–SOS interactions, BI 1701963, is being evaluated for its efficiency alone or in combination with trametinib in solid tumors with KRAS mutation (NCT04111458).

Second, to suppress the activation of RAS, small molecules targeting inactive RAS proteins by a trapping mechanism is an alternative option [91]. ARS853 was identified as a selective inhibitor against the KRAS^G12C^ mutation by covalently reacting with RAS-GDP complex to trap it in its inactive state. ARS853 selectively inhibited downstream signaling and proliferation of cell lines harboring KRAS^G12C^ mutation [92]. Although ARS853 exhibited inhibitory effects in vitro, its poor stability in plasma (t1/2 < 20 min) makes further in vivo study challenging. Considering its potential clinical application, ARS1620 was designed to covalently and selectively react with GDP-bound RAS (RAS-GDP), displaying appropriate pharmacokinetics at the same time. ARS1620 exhibited selective tumor growth repression in a mouse tumor model [93]. Based on ARS1620, a novel-generation KRAS^G12C^ inhibitor, ARS3248 (JNJ-74699157) is undergoing clinical study of its safety and antitumor activity in patients with advanced solid tumors harboring KRAS^G12C^ mutation (NCT04006301). The biochemical mechanism of ARS853 and ARS1620 that possesses potent binding of mutant KRAS protein lies in KRAS-driven catalysis of the reaction between small molecules and Cys12 in the KRAS^G12C^ mutant [94, 95]. Despite ARS853 and ARS1620 suppressing RAS activation, limitations exist because most RAS proteins remain in the GTP-bound conformation. As a result, 2C07 was screened, which could bind in both a nucleotide state and still keep the trapping mechanism of G12C binders [96]. Another chemical probe, BI-2852, which is mechanistically diverse to covalent KRAS^G12C^ inhibitors, was designed to bind with the KRAS pocket with nanomolar affinity [97]. Surprisingly, an objective response has been observed in KRAS^G12C^ lung cancer or colorectal patients when treated with KRAS^G12C^ inhibitors AMG510 and MRTX849 [98, 99]. Regarding another common mutation, KRAS^G12D^, MRTX1133 demonstrated clear tumor regression in KRAS^G12D^ positive preclinical cancer models, including pancreatic adenocarcinoma xenograft models [100].

Third, though targeting the GN binding site has been regarded as unfeasible, a GDP analogue, SML-8–73-1, can form covalent bonds with the GN site and prevent further nucleotide exchange, making targeting the GN site possible. Further, the compound stabilizes GDP-bound KRAS^G12C^, whereas it is not easy to penetrate cells and has limited selectivity [101, 102]. Recently, a KRAS agonist, KRA-533, was identified to suppress mutant KRAS-driven lung cancer in vitro and in vivo, binding the GN binding site to prevent the exchange of GTP to GDP [103].

Fourth, disrupting the RAS effector interaction also represents a direction of RAS inhibition. Kobe0062 and Kobe0065 display inhibitory activity against HRAS and RAF interactions, and they suppress the growth of xenograft tumors harboring KRAS^G12C^ [104]. Moreover, rigosertib was proposed to inhibit RAS signaling as a RAS mimetic to competitively bind to RAS effectors and interfere with their ability to bind to RAS [94].

Considering the essentiality of normal RAS protein, mutant RAS proteins are the focus of drug development, which means the drugs usually target one or several subtypes of RAS mutations. However, a pan-RAS inhibitor, compound 3144, exhibited cellular lethality and tumor growth inhibition without any adverse effects. Compound 3144 was detected to bind to KRAS^G12D^ mutation, wild-type KRAS, NRAS, and HRAS at D38, A59, and Y32. This indicates that pan-RAS inhibitors may have antitumor efficiency and targeting multiple RAS mutations by one compound is feasible [105].

### KRAS^G12C^ inhibitor AMG510

The identification of a cryptic pocket (H95/Y96/Q99) in KRAS^G12C^ enabled the emergence of AMG510, a selective and well-tolerated inhibitor. Its well-tolerability, excellent pharmacological profile and remarkable ability of KRAS^G12C^ tumor repression in vivo encouraged its further clinical study [106]. For the initial evaluable nine patients harboring KRAS^G12C^-mutant cancer treated with AMG510, one patient had a partial response (PR) (NSCLC), six patients had stable disease (SD) (four CRC patients and two NSCLC patients), and two patients had progressive disease (PD) [107]. The additional follow-up in a larger group of patients (59 NSCLC, 42 CRC, 28 other) also exhibited encouraging results, with a 32.2% (19 patients) objective response rate (ORR) and an 88.1% (52 patients) DCR for NSCLC patients with KRAS^G12C^ mutation and a 7.1% (3 patients) ORR and a 73.8% (31 patients) disease control rate (DCR) for CRC patients [15]. The promising results in the subgroup of KRAS^G12C^-mutant NSCLC patients encouraged the multi-center, single-group, open-label, phase 2 trial (CodeBreaK100) of AMG510, administered orally at a dose of 960 mg once daily, in KRAS^G12C^-mutant advanced NSCLC patients who had previously treated with platinum-based chemotherapy or immunotherapy. Among the 124 evaluated patients, there were 46 patients with objective response (37.1%), including in 4 patients (3.2%) with CR and in 42 patients (33.9%) with PR. The DCR was 80.6% in 100 patients. The median duration of response was 11.1 months. The median OS was 12.5 months, and the PFS was 6.8 months. What’s more, the clinical benefit of AMG510 was observed regardless of the mutation status of *TP53, STK11 or KEAP1*, PD-L1 expression level and tumor mutational burden [108]. Based-on the encouraging results of CodeBreaK100 clinical trial, with a 37.1% ORR and 58% of those patients had a duration of response of six months or longer, AMG510 were approved as the first treatment for KRAS^G12C^ mutant NSCLC patients who have received at least one prior systemic therapy. This approval ended the history of undruggable target of RAS in clinic [18]*.* The favorable antitumor efficiency of AMG510 promoted its combination with other targeted or cytotoxic agents and its combinations with MEKi, HERi, EGFRi, PI3Ki, AKTi, SHP2i, and PD-1i resulted in enhanced efficiency in vitro and in vivo [99]. A phase 3 clinical trial compared AMG510 with docetaxel in advanced KRAS^G12C^ mutant NSCLC patients is ongoing (NCT04303780, CodeBreaK200). Further, clinical investigation of AMG510 combined with other targeted agents is also under way (NCT04185883, CodeBreaK101). Notably, clinical acquired resistance to KRAS^G12C^ inhibition has been observed, the mechanisms lie in multiple genomic or histologic mechanisms, in a study investigating mechanisms of the resistance to MRTX849 (adagrasib) monotherapy, in the 17 patients resistant to adagrasib monotherapy, KRAS alterations included G12D/R/V/W, G13D, Q61H, R68S, H95D/Q/R, Y96C, and high-level amplification of the KRAS(G12C) allele were observed, the bypass mechanisms including mutations in NRAS, BRAF, MAP2K1, and RET, loss-of-function mutations in NF1 and PTEN, fusions in ALK, RET, BRAF, RAF1, and FGFR3, histologic transformation [109]. The novel KRAS^Y96D^ mutation affecting the switch-II pocket and polyclonal alterations converging on RAS-MAPK reactivation also represents mechanisms underlying clinical required resistance to KRAS^G12C^ inhibitors [110]. CRC patients possessing KRAS^G12C^ mutation exhibit limited efficiency regarding G12C inhibitors, EGFR signaling was identified as the dominant mechanism of resistance [111]. Novel strategies should be applied to overcome this drug resistance.

### Targeting mutant RAS mRNA

Small interfering RNAs (siRNA) have great clinical potential because of their precise regulation of gene expression. However, the challenge exists in the effective delivery of RNAs to solid tumors. Recently, RNA interference targeting *RAS* has emerged. Systemic delivery of *RAS*-targeted RNAs by nanoparticles, nanoliposomes, or exosomes exhibits anti-proliferative effects in cells and in mouse tumor suppression [112–115]. For example, novel hybrid nanoparticles composed of mutant *KRAS* siRNA, IgG, and poloxamer-188 escaped the clearance of macrophage and delivered the siRNA to cells effectively [114]. Exosomes are essential mediators of cellular communication and have been explored in drug delivery systems because of their endogenous origin [116]. Compared with foreign nanoparticles or liposomes, exosomes do not induce an immune response and avoid clearance by macrophages. Recently, exosomes engineered to load siRNA specific to KRAS^G12D^ successfully inhibited tumors in mouse models of pancreatic cancer and prolonged OS, and further study revealed that CD47 on the surface of exosomes protected them from phagocytosis by monocytes. The relatively higher accumulation of exosomes in tumor tissues is generated by the increased micropinocytosis of tumor cells, which indirectly increases the specificity of exosomes [115]. Chemically modified antisense oligonucleotide (ASO) is also an alternative option for RAS targeting. A 2’-4’ constrained ethyl (cEt)-modified molecule, AZD4785, has good potency with high delivery efficiency and potent *KRAS* knockdown in tumor tissues. Furthermore, because the distribution of AZD4785 does not need any delivery formulation, immune response or clearance is avoided [117]. As a genome-editing system, CRISPR/Cas9 has been successfully utilized to target the oncogenic KRAS^G12S^-mutant allele and induce tumor regression [118].

In addition to the systemic delivery of siRNA, logical siRNA delivery systems represent a strategy. For example, local drug eluteR (LODER) can shed siRNA to peripheral tumor tissue consistently for more than 70 days. As a result, LODER containing KRAS^G12D^-targeted siRNA can suppress the proliferation of pancreatic cancer cells and improve the survival of mice [119]. Another clinical trial also demonstrated siG12D-LODER combination with chemotherapy to be a safe, effective approach for patients with locally advanced pancreatic cancer, with a median OS of 15.12 months. Though the number of enrolled patients was limited to only 15, it indicated that siG12D-LODER has clinical potential [120]. These studies suggest siRNA against mutant *RAS* mRNA represents a promising approach for RAS-targeting therapy.

### Targeting downstream proteins

#### RAF inhibition

The challenge in developing novel drugs targeting RAS directly encourages a focus on the downstream effectors of RAS-driven cancer. The RAF kinases (ARAF, BRAF, and CRAF) constitute essential components in RAS–RAF–MEK–ERK signaling [121]. BRAF inhibitors dabrafenib and vemurafenib evoked notable responses and prolonged the survival of patients with BRAF^V600E^ melanoma by disturbing the enhanced MAPK signaling [122]. However, in KRAS-mutant and RAF wild-type tumors, dabrafenib and vemurafenib activated the MAPK pathway instead of suppressing signaling [123, 124]. The underlying mechanism of this paradoxical activation lies in the activation of CRAF; these BRAF inhibitors drive RAS-dependent BRAF binding to CRAF, initiating the downstream signaling driven by CRAF [125]. Studies implicate that CRAF is vital for mutant KRAS signal transduction and tumor initiation other than BRAF [126]. Recent research found that CRAF-ablated tumors shrank with evidence of apoptosis, whereas there was no reduction in MAPK signaling in tumor tissues [127].

Combined inhibition of EGFR and CRAF also effectively suppresses the growth of patient-derived xenograft models with KRAS mutation [128]. A preclinical study also demonstrated that pan-RAF inhibitors RAF709 or LY3009120 exhibited antitumor activity in vitro and in vivo [129, 130]. Recently, a phase I clinical study of lifirafenib (BGB-283), a RAF family kinase inhibitor, showed patients with KRAS-mutated endometrial cancer and NSCLC had a confirmed PR (n = 1 each), while no response was observed in patients with KRAS- or NRAS-mutated colorectal cancer (n = 20) [131]. This evidence suggests that CRAF is a rational drug target and that pan-RAF inhibitors have the potential for RAS-mutant tumors.

### MEK inhibition

The MEK inhibitor trametinib exhibits improved PFS and OS among patients who had metastatic melanoma with BRAF^V600E^ or BRAF^V600K^ mutation [132], indicating that MEK inhibition may represent an alternative strategy of halting the preternatural MAPK signaling in tumor. Furthermore, regarding NRAS-mutant melanoma patients, MEK inhibitors show activity [133, 134]. In a phase II study, six of 30 NRAS-mutant patients showed a PR to MEK162, a small molecule MEK inhibitor [133], and binimetinib improved the PFS of NRAS-mutant patients compared with dacarbazine (2.8 months vs 1.5 months, *P* < 0.001) [134]. However, a series of clinical studies showed no significance for MEK inhibition regarding KRAS-mutant tumors [135–138]. The underlying mechanism of this resistance was considered to be the reactivation of MEK. MEK inhibition is thought to relieve the feedback suppression of upstream signaling. Furthermore, CRAF mediates the reactivation of MAPK signaling [139, 140]. Thus, the notion of co-targeting MEK and CRAF or pan-RAF emerged; additionally, the combination strategy exhibited better anti-proliferation of cancer cells harboring KRAS mutations compared with MEK inhibitors alone [139, 140]. In a phase II clinical trial, combination of the MEK inhibitor refametinib plus sorafenib, both multiple kinase inhibitors that inhibit CRAF, showed antitumor activity in HCC patients, especially those with KRAS mutations [141]. The dual MEK/RAF inhibitor RO5126766(VS-6766) exhibited antitumor activity in participants harboring RAS mutations; one patient with an NRAS mutation obtained a PR [142]. The subsequent evaluation of RO5126766 in solid tumors or multiple myeloma (12 NSCLC, five gynecological malignancy, four colorectal cancer, one melanoma, and seven multiple myeloma) with RAS–RAF–MEK pathway mutations showed that 7 of 26 evaluable patients achieved objective responses [143]. Remarkedly, the combination of VS-6766 with defactinib obtained 70% ORR (7 of 10 evaluable patients) in LGSOC patients harboring KRAS mutation [17]. Further clinical studies of VS-6766 for KRAS-mutant NSCLC patients are ongoing (NCT03681483 and NCT03875820).

Because of the absence of paradoxical activation and the resistance to single MEK inhibitors for RAS-mutant tumors, MEK inhibitors are considered candidates for combination in RAS-mutant tumors and have exhibited feasible antitumor activity in vitro and in vivo [144–150]. For example, co-targeting of anti-apoptotic proteins BCL-XL or MCL-1 and MEK promotes tumor regression in KRAS-mutant tumor models compared with MEK targeting alone [144, 145]. Serine threonine phosphatase PP2A inhibition could confer MEK inhibitor resistance in KRAS-mutant lung cancer cells [148]. Moreover, combined insulin-like growth factor 1 receptor (IGF1R) and MEK blockade showed significant effects in KRAS-mutant lung cancer cells and in KRAS-driven mice tumor models [146]. Combined treatment with poly (adenosine diphosphate–ribose) polymerase (PARP) inhibitors and MEK inhibitors elicited synergistic effects in vitro and in vivo in multiple RAS mutant tumor models [150]. Remarkably, a phase II study of docetaxel and trametinib (MEK inhibitor) in NSCLC patients with KRAS mutation exhibited a 33% RR and a median survival of 11.1 months [151]. KRAS mutations may alter the expression of immune inhibitory molecules or immune cell infiltration, which indicates that MAPK signaling may have an impact on immune therapy [58, 59]. Combination of MEK inhibition and PD1/PD-L1 blockade prolonged OS of a KRAS-driven lung cancer model [152]. In addition, combining MEK inhibitors with agonist antibodies targeting the immunostimulatory CD40 receptor resulted in synergistic antitumor efficacy in KRAS-driven tumors [153].

### ERK inhibition

The ERK inhibition strategy exhibits therapeutic potential against RAS-mutant, BRAF-mutant, BRAF- or MEK-inhibitor resistant tumors [154–156]. A novel molecule selectively targeting ERK, SCH772984, induced tumor regression in mouse xenograft models with KRAS or NRAS mutations [154]. AZD0364 exhibited dose- and time-dependent modulation of ERK1/2-dependent signaling to result in tumor regression in sensitive BRAF-and KRAS-mutant xenografts [157, 158]. Another similar small molecule, BVD523 (ulixertinib), exhibited antitumor activity for MEK–BRAF in concurrent or single targeting in resistant models in vitro or in vivo [156]. A clinical trial assessing BVD523 provided the first clinical evidence that ERK inhibitors were effective for patients with NRAS mutations, in which three of 18 NRAS-mutant patients responded to BVD523 [159]. As a result, ERK inhibition may represent a potential clinical weapon regarding RAS-mutant tumors [160].

### PI3K–AKT–mTOR inhibition

The PI3K–AKT–mTOR pathway represents another signaling pathway induced by RAS, it may serve as a complementary role for the RAF–MEK–ERK cascade [161]. As a result, co-targeting of the MAPK and PI3K–AKT–mTOR pathways was developed in preclinical trials. Typically, combination of PI3K and MEK inhibitors displayed synergistic effects in suppressing the proliferation of RAS-mutant cells and regressing xenografted RAS-mutant tumors [162–167]. For example, a dual pan-PI3K and mTOR inhibitor, NVP-BEZ235, was synthetic with MEK inhibitor in repressing KRAS^G12D^ mutant lung cancers [163]. Combination of MEK and PI3K/mTOR1,2 inhibition could induce apoptosis in NRAS mutant melanoma cancer cells and shrink tumor in mouse xenograft model [162]. The combination of KRAS^G12C^ inhibitor ARS1620 plus PI3K inhibitors was effective in vitro and in vivo including patient-derived xenografts models for NSCLC models with KRAS^G12C^ mutation [165].

With the rational combination strategy and validated pre-clinical efficiency. However, in clinical trials, the combination of PI3K and MEK inhibitors exhibited poorly tolerated toxicities or limited efficiency, which limited their utility in the clinic [168–173]. For example, there was no response in 23 RAS-mutant acute myeloid leukemia patients receiving combined MEK and AKT inhibition [174]. In 57 patients with solid tumors harboring RAS/RAF/PI3K mutations, the combination of GSK2126458, a pan-PI3K/mTOR inhibitor, with MEK inhibitor exhibited limited efficiency. The skin and gastrointestinal toxicities were poorly tolerated [168]. Similarly, 89 patients with RAS/RAF mutations were enrolled to study the efficiency and safety of MEK inhibitor binimetinib plus PI3K inhibitor buparlisib, only 6 patients achieved partial response and 32/89 patients suspended treatment due to the serious adverse events [173]. In addition, as a result, further investigations of the proper dosing schedule or more selective inhibitors are needed.

Similarly, mTOR inhibitor or its combination with other targets inhibitors, such as HDAC, BCL-2/BCL-XL, WEE1, KRAS, and MEK, exhibited inhibitory effects for RAS-driven tumors in vitro and in vivo [175–179]. For example, combined mTOR and HDAC inhibitors resulted in tumor regression in a mice xenografts models of *KRAS*-mutant NSCLC in vivo [178]. WEE1 and mTOR inhibitor induced efficient apoptosis in *KRAS*-mutant NSCLC cell lines and suppressed tumor growth in mice model [177]. A mTOR inhibitor, AZD8055 with a BCL-2 inhibitor exhibited synergistic cytotoxic effects in *KRAS*-mutant colorectal cancer cells [176]. However, the mTOR inhibitors exhibited limited efficiency against cancers harboring *RAS* mutation in clinical trials [180–185]. For instance, the phase II study of evaluating the efficiency of Everolimus, an mTOR inhibitor in metastatic colorectal adenocarcinoma previously treated with chemotherapy, among the 100 patients receiving daily everolimus, those with *KRAS* mutation (41 patients) owned shorter OS (5.59 months vs 7.06 months) and lower DCR (7% vs 14%) compared with those with wild *KRAS* [181]. Similarly, in a cohort of cancer patients receiving everolimus, only 1/12 patients with *KRAS* mutation had disease control, while 15/31 wild cases benefited from the treatment [186].

Remarkedly, the combination of MEK and AKT inhibitors obtained antitumor ability in certain *KRAS-*driven human cancers, in a cohort of patients with solid tumors receiving MEK1/2 inhibitor selumetinib and allosteric AKT inhibitors MK-2206, 3 of 13(23%) NSCLC patients and 1 of 2(50%) OVCA patients with *KRAS* mutation obtained PR, while there was no objective response in colorectal cancers with KRAS mutations [167]. In *RAS-*mutant AML, combined MEK and AKT inhibition had no clinical efficiency in 23 patients with *RAS* mutation [174]. Further investigations of the combination of MEK and AKT inhibitors in clinic are needed.

### Targeting metabolic processes affected by RAS mutations

It is essential for tumors to reprogram the metabolic processes in support of the elevated proliferation state. Oncogenic RAS-driven cancer cells are known to refer to elevated macro-pinocytosis and macro-autophagy. Furthermore, increased glucose metabolism and dependency on glutamine are also hallmarks for RAS-mutant tumors [161]. In PDAC, autophagy plays a critical role for tumor growth and progression [187, 188]. It was found that MAPK signaling and autophagy pathways cooperate to promote RAS-mutant cell survival [189]. Thus, the autophagy inhibitor chloroquine was combined with the ERK inhibitor SCH772984, resulting in elevated antitumor activity in vitro and in vivo in PDAC [190]. Furthermore, combined inhibition of MEK plus autophagy showed synergistic antitumor activity against patient-derived xenografts of KRAS-mutant PDAC and NRAS-mutant melanoma [191]. These data suggest the strategies of combining autophagy blockade and MAPK inhibition may represent new avenues for targeting RAS-mutant tumors.

#### Other strategies of targeting mutant RAS

PROteolysis TArgeting Chimeras (PROTACs) has emerged a novel and promising strategy to eliminate a protein of interest (POI). Bifunctional molecules combine POI with an E3 ligase, forming a ternary complex, enabling E3 ligase to ubiquitinate the POI and subsequently the POI is recognized and degraded [192, 193]. Recently, a bifunctional molecule, LC-2, was reported to covalently binds KRAS^G12C^ with a MRTX849 bridge and recruits E3 ligase. Subsequently, the KRAS^G12C^ protein was ubiquitinated and degraded persistently, the MAPK signaling was suppressed in cancer cells [194]. Similarly, PROTACs incorporating ARS-1620 and E3 ligase through a thalidomide scaffold could degraded GFP-KRAS^G12C^ in reporter cells [195]. PROTACs represent a novel direction in small-molecule-mediated targeting and degradation of RAS. However, it depends on the development of bifunctional molecular binding to target protein directly. Therefore, tag-based PROTACs have been developed, which utilizes the CRISPR/Cas-mediated locus-specific knock-in or transgene expression to form the fusion of tag protein and POI, small molecules subsequently induce the degradation of tag fusion protein [192]. The tag-based PROTACs mainly contain the haloPROTACs system and the dTAG system [196]. In the haloPROTACs system, the administration of HyT13 successfully degraded haloTag–HRAS^G12V^ fusion protein in NIH-3T3 cells and suppressed the tumor formation in mice [197]. Similarly, the dTAG system, which relies on the fusion of FKBP12^F36V^ to the terminus of POI, effectively degraded FKBP12^F36V^-tagged KRAS^G12V^ and decreased the downstream signaling in cells [198]. What’s more, taking advantage of haloPROTACs, a ligand-inducible tractable affinity-directed protein missile system (L-AdPROM), in which aHRAS conjugated to the Halo-tag and tagged with a FLAG reporter, successfully degraded RAS and reduce the RAS-driven signaling in A549 cells [199]. In addition, PROTACs regarding some targets in RAS signaling pathway, such as MEK, PDEδ, TBK1 and SHP2, also successfully degraded the corresponding targets and suppressed the RAS signaling in vitro or in vivo [200–203].

Mutant RAS may generate abnormal proteins that can evoke the immune response, suggesting the feasibility of immunotherapy utilization in RAS-mutant cancer. One study noted the shrinkage of all lung metastases (seven in total) after the transfer of KRAS^G12D^-specific CD8 + T cells in a mCRC patient, which indicated immunotherapy may have potential in targeting mutant KRAS [204]. Several clinical studies are ongoing to evaluate the efficiency and safety of immunotherapy targeting mutant RAS. For example, peripheral blood lymphocytes with modified mTCR that target KRAS^G12D^ and KRAS^G12V^ are under clinic investigation for rectal and pancreatic cancer (NCT03745326 and NCT03190941, respectively). In addition, a mRNA-based cancer vaccine (V941) targeting the most commonly occurring KRAS mutations (G12D, G12V, and G12C) is under clinical study (NCT03948763).

Another strategy targeting mutant RAS is screening for synthetic lethal interactors, which aims to identify genes that are vital to RAS-mutant but not wild-type cells. The progress of synthetic lethal interactors for mutant RAS is reviewed elsewhere [14].

## Conclusion

Great progress has been made in the past few years, especially with the approval of Lumakras (sotorasib, AMG510) in treatment of KRAS^G12C^-mutant NSCLC patients who have received at least one prior systemic therapy, this approval ended the history of no drug in clinic for RAS mutation. However, there are limited patients who can benefit from it, with only about 13% of KRAS^G12C^ mutation in NSCLC patients, CRC patients with KRAS^G12C^ mutation obtained low clinical response [18]. Thus, further investigation of strategies targeting mutant RAS is necessary. One potential direction is the combination of several inhibitors. These combination strategies are designed to avoid reactivating the MAPK pathway, among which, MEK inhibitors represent the most favorable candidate for combination because of the absence of paradoxical activation and the existence of approved MEK inhibitors. Apart from combining molecules in the MAPK or PI3K–AKT cascades with MEK inhibitors, screening genes that sensitize MEK inhibitors based on short-hairpin RNA or CRISPR may also identify potential combinable candidates [145, 147, 205]. MRTX 849, RMC-4630 and VS-6766 had early encouraging outcomes, demonstrating antitumor activity in patients harboring KRAS mutation (MRTX 849 for KRAS^G12C^) [16, 98, 99, 143]. However, the efficacy and safety still need to be confirmed through large samples and multi-center phase III clinical studies before clinical application. RNA interference represents a promising approach to suppress the expression of mutant RAS, but clinical studies are needed to evaluate their efficiency and safety. The issues of targeting RAS are still ongoing, and we must recognize that a simple therapy will not be effective for all RAS-mutant cancers. Consequently, multiple RAS-targeting strategies are needed for RAS-mutant subsets. Targeting mutant RAS remains a potentially effective treatment in the future.

## Data Availability

Not applicable.

## Abbreviations

aBTC: Advanced biliary tract cancer
AJCC: American joint committee on cancer
AML: Acute myeloid leukemia
aPC: Advanced pancreatic cancer
ASO: Antisense oligonucleotide
anti-EGFR: Anti-epidermal growth factor receptor
BSC: Best supportive care
Cap: Capecitabine
CC: Colon cancer
CMML: Chronic myelomonocytic leukemia
CP: Carboplatin and paclitaxel
CRC: Colorectal cancer
CTLA-4: Cytotoxic lymphocyte antigen 4
DCR: Disease control rate
EC: Endometrial cancer
EOC: Epithelial ovarian cancer
ESC: Esophageal carcinoma
FOLFIRI: 5-Fluorouracil, folinic acid, and irinotecan
FOLFOX4: Oxaliplatin, fluorouracil, and leucovorin
FTIs: FTase inhibitors
FU/LV: 5FU + leucovorin
GC: Gastric cancer
Gem: Gemcitabine
GICA: Gastrointestinal cancer
GGTase: Geranylgeranyltransferases
GN: Guanine nucleotide
HVRs: Hypervariable regions
ICB: Immune checkpoint blockade
IFL: Fluorouracil [5-FU], leucovorin, and irinotecan
IGF1R: Insulin-like growth factor 1 receptor
IMA: Invasive mucinous adenocarcinoma of the lung
KMP: KRAS-mutant patients
KWP: Wild-type KRAS patients
LAPC: Locally advanced pancreatic cancer
lnc: Include, means including patients with RAS mutation
LODER: Local drug eluteR
LUAC: Lung adenocarcinoma
mCRC: Metastatic colorectal cancer
MM: Malignant melanoma
MSI: Microsatellite instability
Mut: Mutation
NA: None
Napa: Nanoparticle
ND: Not determined
NF1: Neurofibromatosis type 1
NM: Not mentioned
NMP: NRAS-mutant patients
NSCLC: Non-small-cell lung carcinoma;
OEC: Ovarian epithelial cancer
OS: Overall survival
OVCA: Ovarian cancer
Ox: Oxaliplatin
PanIN: Pancreatic intra-epithelial neoplasia
PanNEN-G3: Pancreatic neuroendocrine neoplasm grade-3
PBL: Peripheral blood lymphocytes
PC: Pancreatic cancer
PD: Progressive disease
PD1: Programmed cell death protein 1
PDAC: Pancreatic duct adenocarcinoma
PDEδ: Phenyl-binding protein phosphodiesterase δ
PD-L1: Programmed cell death 1 ligand 1
PR: Progesterone receptor
PTPN11: Tyrosine-protein phosphatase non-receptor type 11
RalGEFs: Ral guanine exchange factors
RC: Rectal cancer
RFS: Recurrence-free survival
RNAi: RNA interference
SD: Stable disease
SIA: Small intestinal adenocarcinoma
SHP2: SRC homology-2-containing protein tyrosine phosphatase 2
TIL: Tumor-infiltrating lymphocyte
